# Postpartum endometritis and infection following incomplete or complete abortion: Case definition & guidelines for data collection, analysis, and presentation of maternal immunization safety data

**DOI:** 10.1016/j.vaccine.2019.09.101

**Published:** 2019-12-10

**Authors:** C.E. Rouse, L.O. Eckert, F.M. Muñoz, J.S.A. Stringer, S. Kochhar, L. Bartlett, M. Sanicas, D.J. Dudley, D.M. Harper, M. Bittaye, L. Meller, F. Jehan, H.C. Maltezou, M. Šubelj, A. Bardaji, A. Kachikis, R. Beigi, M.G. Gravett, for the Global Alignment of Immunization Safety in Pregnancy (GAIA) Postpartum Endometritis, Infection following Incomplete or Complete Abortion Work Group

**Affiliations:** a Department of Obstetrics and Gynecology, Indiana University, Indianapolis, IN, USA; b Departments of Obstetrics and Gynecology and Global Health, University of Washington, Seattle, WA, USA; c Department of Pediatrics, Section on Infectious Diseases, Baylor College of Medicine, Houston, TX, USA; d Department of Obstetrics and Gynecology, University of North Carolina, Chapel Hill, NC, USA; e Global Healthcare Consulting; University of Washington, Seattle, USA; f Erasmus MC, University Medical Center, Rotterdam, the Netherlands; g Department of International Health, Johns Hopkins University, Baltimore, MD, USA; h Sanofi Pasteur, Asia and JPAC Region, Singapore; i University of Virginia, Department of Obstetrics and Gynecology, Charlottesville, VA, USA; j University of Michigan, Departments of Family Medicine and Obstetrics and Gynecology, Department of Epidemiology, Ann Arbor, MI, USA; k Edward Francis Small Teaching Hospital/University of The Gambia and Medical Research Council, The Gambia at London School of Hygiene and Tropical Medicine, USA; l Safety & Pharmacovigilance, Syneos Health, Raleigh, NC, USA; m Department of Pediatrics and Child Health, Aga Khan University, Karachi, Pakistan; n Department for Interventions in Healthcare Facilities, Hellenic Center for Disease Control and Prevention, Athens, Greece; o National Institute of Public Health, Ljubljana, Slovenia; p Barcelona Institute for Global Health, Barcelona, Spain; q Department of Obstetrics and Gynecology and Global Health, University of Washington, Seattle, WA, USA; r University of Pittsburgh School of Medicine, Pittsburgh, PA, USA

**Keywords:** Postpartum endometritis, Sepsis, Septic abortion, Adverse event, Immunization, Guidelines, Case definition

## 1 Preamble

### 1.1 Need for developing case definitions and guidelines for data collection, analysis, and presentation for postpartum endometritis and infection following incomplete or complete abortion as adverse events following maternal immunization

**Fig. 1:**
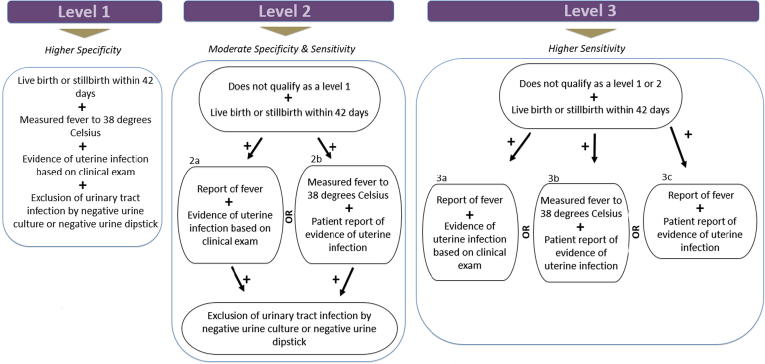
Postpartum endometritis diagnostic levels of certainty.

**Fig. 2:**
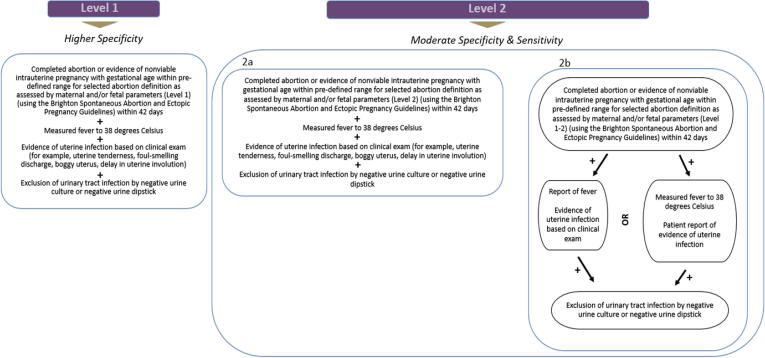
Infection following incomplete or complete abortion, diagnostic levels of certainty 1 and 2.

**Fig. 3:**
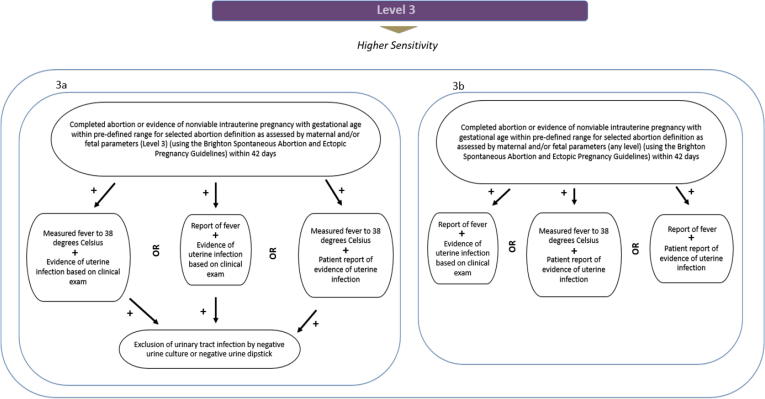
Infection following incomplete or complete abortion, diagnostic level of certainty 3.

Remarkable progress has been made in the implementation of vaccinations against infectious diseases worldwide. Immunization of pregnant women is important because pregnancy is thought to modulate the immune system to tolerate a growing fetus, and this, along with the physiologic changes of pregnancy, may increase susceptibility to certain infectious diseases [1]. Immunizing the mother also provides direct protection via transplacental transfer of antibodies for the fetus during pregnancy and for the neonate following delivery. Pregnancy outcomes related to the administration of immunizations during pregnancy, however, have been less well studied. In particular, puerperal sepsis (infection of the female genital tract following childbirth or abortion/miscarriage) has not been well studied following maternal immunization. Puerperal sepsis is responsible for over 10% of maternal deaths worldwide and disproportionately occur in low- and middle-income countries (LMICs) [2], [3]. Puerperal sepsis is defined by the World Health Organization (WHO) as infection of the genital tract occurring any time between the rupture of membranes or labor and the 42nd day postpartum. This definition encompasses both chorioamnionitis and postpartum endometritis or endomyometritis (PPE), two of the most common infections surrounding childbirth [4]. These complications are likely to be inconsistently reported. ICD-10 codes for “sepsis following incomplete or complete abortion” (O03.87) and “endometritis following delivery” (O86.12) do not include any diagnostic criteria (see Fig. 1, Fig. 2, Fig. 3).

Early identification and appropriate management of these infections is essential for prevention of maternal and infant morbidity and mortality. Efforts have been made to standardize the definitions for maternal infectious conditions or sepsis in order to improve identification of the condition, facilitating timely treatment based upon prompt identification, and assessment of the burden of maternal puerperal sepsis across settings [5], [6].

While infectious complications following delivery or abortion are important pregnancy outcomes and should be reported in vaccine trials, the variability in terms used and lack of standardized diagnostic criteria make interpretation of available data challenging. Our aim is to provide guidance for the appropriate diagnosis of postpartum endometritis (PPE) in studies of maternal vaccination and to improve data quality by harmonizing the definitions, allowing comparability across studies. In addition to PPE, we have also included levels of evidence for diagnostic criteria for septic abortion, since the clinical signs and symptoms and the microbial pathogens are similar to those of PPE [7]. The distinction between the PPE and septic abortion is frequently based solely upon the gestational age at the time the pregnancy concludes. Chorioamnionitis, or intra-amniotic infection, is discussed in a related article [8].

#### 1.1.1 Postpartum endometritis (PPE)

##### 1.1.1.1 Incidence, microbiology and risk factors for postpartum endometritis

Infection following childbirth occurs commonly. Although the incidence of puerperal sepsis, including both PPE and chorioamnionitis, varies widely worldwide, estimates range from less than 1–10% [9], [10]. As with maternal mortality in general, death and morbidity from puerperal sepsis are more common in low-resource settings compared to high-resource settings [2]. Among deaths attributed to puerperal sepsis, PPE is the most frequent cause of death in the 3–7 days following delivery [11].

Postpartum endometritis is one of the primary causes of maternal infection following delivery, occurring after 1–3% of vaginal births and in up to 27% of cesarean deliveries [12]. Other common causes of postpartum febrile morbidity include mastitis, urinary tract infection, and abdominal or perineal wound infection. The relative frequency of these infections seems to vary by clinical setting. Mastitis (3–4.5%), followed by urinary tract infection (2.4–3%) and endometritis (1.7–2%) were the most common postpartum infections identified in an observational study in Sweden [13]. A prospective study in Uganda found that endometritis was the most common in-hospital postpartum infection (1.8%) [14].

Postpartum endometritis is an infection of the decidua, or uterine lining. Because the myometrial, or muscular layer, is also often involved in postpartum uterine infection, the term “endomyometritis” is often used to describe the infection. It is typically polymicrobial, involving both facultative and anaerobic bacteria; genital mycoplasmas and sexually transmitted organisms such at *C. trachomatis* have also been found in endometrial biopsy specimens of patients with endometritis [15]. The microbiology of PPE found in low- and middle-income countries (LMIC) differs from that found in high-income countries, with organisms such as *E. coli*, *Proteus spp*, *N. gonorrhoea*, and *S. pneumonia* isolated most frequently in LMIC [16]. Group B *Streptococcus*, frequently found in the genital tracts of pregnant women, is thought to be responsible for a significant number of infections in postpartum women worldwide [17]. Group A *Streptococcus* infection, while relatively rare, is associated with a particularly rapid and severe course of postpartum endometritis and with rapidly progressive soft tissue infections [18]. Overall mortality approaches 20% in cases of Group A *Streptococcus*, and increases to 60% if septic shock is present [19], [20], [21].

The most important risk factor for postpartum endometritis is cesarean delivery. This risk is 21 times higher when the cesarean occurs following labor [22]. Other risk factors include maternal BMI > 35 kg/m^2^, prolonged rupture of membranes, chorioamnionitis, lack of antibiotic prophylaxis, poor nutrition, location of delivery, low socioeconomic status and multiple vaginal examinations [10], [12], [18], [23], [24].

##### 1.1.1.2 Diagnosis of postpartum endometritis

Postpartum endometritis is a clinical diagnosis, usually defined as maternal pyrexia together with physical signs of endometrial infection. Clinical signs and symptoms usually include fever and one or more of the following:uterine tenderness, abnormal vaginal discharge or odor, and delay in the rate of reduction of size of the uterus [25]. Other laboratory evidence is often used to support the diagnosis of infection, such as an elevated white blood cell count; an elevated blood lactic acid concentration is useful in identifying hypoperfusion related to sepsis. While detection of bacteremia with blood cultures can be useful to tailor antibiotic treatment in complicated infections (for example, in immunocompromised patients), the polymicrobial nature of most infections limits its utility; thus, in most patients with uncomplicated infection, broad-spectrum antibiotic treatment is recommended and usually effective [26], [27]. Abnormal vital signs consistent with sepsis physiology (such as elevated or depressed temperature, hypotension, tachycardia, tachypnea or bradypnea, hypoxia, leukocytosis or leukopenia, bandemia (excess immature white blood cells), and lactic acidosis) can help identify women requiring urgent evaluation and treatment [28], [29]. Endometrial bacterial cultures are challenging to obtain without contamination [30] and rarely impact treatment decisions; thus they are not routinely performed in most settings [31]. One exception may be Group A *Streptococcus*, which remains extremely sensitive to penicillin.

#### 1.1.2 Infection following incomplete or complete abortion

##### 1.1.2.1 Incidence, microbiology and risk factors for infection following incomplete or complete abortion

Abortion is a common pregnancy outcome. Spontaneous abortion, or pregnancy loss prior to 22 weeks gestation, has been described in a related document [32]. The cumulative risk of infection following abortion between weeks 5 and 20 of pregnancy ranged from 11% to 22% in one systematic review [33]. The rate of infection following spontaneous abortion is low.

Induced abortion is the termination of pregnancy through medical or surgical procedures. Approximately 25% of pregnancies end in induced abortion worldwide, and the annual rate of abortion for reproductive age women is approximately 35 per 1000[34]. In high-income countries (HIC) with legal abortion, the rate of fatal sepsis following medically induced abortion is very low (<1/10,000) [35], with recent studies reporting an overall incidence of infection in this population of 0.1–0.5% [36], [37]. In women in HIC undergoing surgical abortion procedures (i.e., dilation and curettage), the rate of infection is reported to be 0.0–0.4% [37], [38]. Worldwide, the rate of infection after surgical abortion is 0.1–4.7% [39]. Rates of infection related to abortion in LMIC is difficult to ascertain, but given that almost all unsafe abortions occur in developing countries [40], this statistic is likely to be higher than in HIC.

Infection following incomplete or complete abortion (induced or spontaneous), results from infection of the products of conception retained within the uterine cavity. While most bacteria that cause infection following abortion are genital flora (e.g. Group B streptococcus, *B. fragilis, E. coli*), anaerobic bacteria are particularly associated with the condition and found in 60% of women with positive blood cultures; bacteria causing genital infection have also been implicated in infection following abortion (*N. gonorrhoea, C. trachomatis, Trichomonas*) [41], [42], [43]. *C. perfringens* and Group A streptococcus are rare isolates in these cases, but are particularly virulent secondary to their ability to produce toxins [41], [44], [45].

Uterine instrumentation increases the risk of infection following abortion [7]. Retained products of conception provide a nidus for the development of infection. Many studies report an increased incidence of post-abortion complications in settings where abortion laws are restrictive [46], [47], [48]. Treatment delays are thought to contribute significantly to mortality associated with induced abortion [7]. Unsafe/unsterile conditions in which any abortion (regardless of presence of fetal cardiac activity) is performed also increase the risk of infection.

##### 1.1.2.2 Diagnosis of infection following incomplete or complete abortion

As with postpartum endometritis, infection following incomplete or complete abortion is a clinical diagnosis in a patient with an incomplete abortion or following a completed abortion in which there is pyrexia and evidence of uterine tenderness. Peritonitis may be present. Retained products of conception, purulent discharge and vaginal bleeding are common signs associated with the condition. While not required for the diagnosis or prior to treatment, culture data is often obtained; products of conception should be sent for culture and Gram stain, if available [49].

### 1.2 Methods for the development of the case definition and guidelines for data collection, analysis, and presentation for postpartum endometritis and infection following incomplete or complete abortion as adverse events following immunization during pregnancy

Following the process described in the overview paper [50] as well as on the Brighton Collaboration Website http://www.brightoncollaboration.org/internet/en/index/process.html, the Brighton Collaboration Postpartum Endometritis and Sepsis/Infection after Abortion *Working Group* was formed in 2018 and included members of clinical, academic, public health, research and industry background. The composition of the working and reference group as well as results of the web-based survey completed by the reference group with subsequent discussions in the working group can be viewed at: http://www.brightoncollaboration.org/internet/en/index/working_groups.html.

To guide the decision-making for the case definition and guidelines, a literature search was performed using Medline, Embase and the Cochrane Libraries, including the terms “endometritis,” “postpartum,” “postpartum sepsis,” “septic abortion,” “abortion,” “postpartum inflammation,” and “postpartum fever*.*” Exhaustive search strategies were implemented using appropriate keywords, accepted MeSH words, and combinations thereof. All abstracts were screened for possible reports of abortion following immunization. Searches were restricted to references in English, and involving only human subjects. Multiple general medical, pediatric, obstetrics and infectious disease textbooks were also searched.

The search and screening resulted in the identification of articles with potentially relevant material for further evaluation. This literature provided several different general definitions for PPE and infection following abortion, their epidemiology, numerous descriptions for causes and/or risk factors and the diagnostic criteria put forth. Most publications addressing postpartum endometritis and infection following abortion following immunization were case reports of single cases or case series describing various pregnancy outcomes, for which terminology was very inconsistent and very few used case definitions.

### 1.3 Rationale for selected decisions about the case definition of postpartum endometritis and infection following incomplete or complete abortion as adverse events following immunization during pregnancy

#### 1.3.1 Related term(s)

Postpartum endomyometritis is a term that is increasingly utilized to describe uterine infection after childbirth in order to highlight that the infection does not solely involve the endometrial layer of the uterus, but also the myometrial, or muscular layer.

There are several terms in use to describe sepsis or infection after abortion, including “septic abortion,” “post-abortion infection,” and “infection after miscarriage or abortion.” For the purposes of this document, we will use the terms “postpartum endometritis,” and “infection following incomplete or complete abortion.”

#### 1.3.2 Formulating a case definition that reflects diagnostic certainty: Weighing specificity versus sensitivity

It needs to be re-emphasized that the grading of definition levels is entirely concerned with diagnostic certainty, not clinical severity of an event. Thus, a clinically very severe event may appropriately be classified as Level Two or Three rather than Level One if it could reasonably be non-abortion related. Detailed information about the severity of the event should always be recorded, as specified by the data collection guidelines.

The number of symptoms and/or signs that will be documented for each case may vary considerably. Our case definition has been formulated such that the Level One definition is highly specific for the condition. As maximum specificity normally implies a loss of sensitivity, two additional diagnostic levels have been included in the definition, offering a stepwise increase of sensitivity from Level One down to Level Three, while retaining an acceptable level of specificity at each level. In this way it is hoped that all possible cases of post-partum endometritis and infection following incomplete or complete abortion can be captured.

#### 1.3.3 Rationale for individual criteria or decision made related to the case definition

There is a need to consider data sources and availability of existing data when defining pregnancy outcomes in research. The interpretation of data is difficult when definitions of commonly used terms differ in the literature. Flexibility and alignment with existing definitions where studies/surveillance are performed are necessary to ensure comparability and interpretation of data. Sometimes these data are not made available.

#### 1.3.4 Determination of the gestational age at onset of the event

Proposed algorithms for defining abortion, fever and for estimating gestational age for studies in various settings are presented in related manuscripts [32], [51], [52]. We propose utilizing these algorithms when reporting cases of postpartum endometritis or infection/sepsis following abortion following vaccine administration.

#### 1.3.5 Determination of fever

Maternal fever is defined as the endogenous elevation of at least one measured body temperature of ≥38 °C, as measured by any validated device [52].

#### 1.3.6 Gestational age cut-offs for definition of sepsis/infection after abortion

The gestational age used to define second trimester abortion varies widely among resource settings. However, we have chosen to utilize the GAIA project definition of abortion using the Brighton guidelines in order to ensure harmonization across GAIA definitions, resulting in the fewest number of missed cases of post-partum endometritis and infection after complete or incomplete abortion as possible. The GAIA definition of a spontaneous abortion is a pregnancy loss that occurs up to 21 weeks 6 days, with outcomes after that gestational age pertaining to the stillbirth or preterm birth categories. We emphasize that this gestational age cut-off should be used for research and data collection purposes only, and is not intended to inform or impact clinical care. Intrauterine infection diagnosed after 21 weeks 6 days will pertain to the GAIA definition of Chorioamnionitis [8].

#### 1.3.7 Timing post immunization in pregnancy

The time interval from immunization to onset of postpartum endometritis or sepsis/infection after abortion is not part of the definition, but it is recommended to be used in the data analysis to examine factors such as temporal clusters as well as determining whether the outcome of interest occurred before or after the vaccine exposure. Where feasible, details of this interval should be assessed and reported as described in the data collection guidelines (see guideline 34, Section 3.2).

### 1.4 Guidelines for data collection, analysis and presentation

As mentioned in the overview paper [50], the case definition is accompanied by guidelines that are structured according to the steps of conducting a clinical trial, i.e. data collection, analysis and presentation. Neither case definition nor guidelines are intended to guide or establish criteria for management of ill infants, children, or adults, but were instead developed to improve data comparability.

### 1.5 Periodic review

Similar to all Brighton Collaboration case definitions and guidelines, review of the definition with its guidelines is planned on a regular basis (i.e. every three to five years) or more often if needed.

## 2 Case definition of postpartum endometritis and infection after incomplete or complete abortion and ascertainment of levels of certainty

### 2.1 Postpartum endometritis

#### 2.1.1 Level 1 (Highest level, gold standard diagnosis, highest specificity)

**Table:** 

Live birth or stillbirth within 42 days
AND
Measured fever to 38 degrees Celsius
AND
Evidence of uterine infection based on clinical findings that may include uterine tenderness, pelvic pain, abnormal vaginal discharge or odor, and delay in the rate of reduction of size of the pospartum uterus
AND
Exclusion of urinary tract infection by negative urine culture or negative urine culture

#### 2.1.2 Level 2 (Missing at least one confirmatory diagnostic parameter; moderate sensitivity and specificity)

**Table:** 

2A
Live birth or stillbirth within 42 days
AND
Patient report of fever
AND
Evidence of uterine infection based on clinical exam
AND
Exclusion of urinary tract infection by negative urine culture or negative urine culture
2B
Live birth or stillbirth within 42 days
AND
Measured fever to 38 degrees Celsius
AND
Patient report of evidence of symptoms consistent with uterine infection (e.g., pelvic pain, uterine tenderness, abnormal vaginal discharge or odor)
AND
Exclusion of urinary tract infection by negative urine culture or negative urine culture

#### 2.1.3 Level 3 (Less specificity)

**Table:** 

3A
Live birth or stillbirth within 42 days
AND
Patient report of fever
AND
Evidence of uterine infection based on clinical exam
3B
Live birth or stillbirth within 42 days
AND
Measured fever to 38 degrees Celsius
AND
Patient reports symptoms consistent with uterine infection, as outlined above
3C
Live birth or stillbirth within 42 days
AND
Patient report of fever
AND
Patient report symptoms consistent with uterine infection, as outlined above

### 2.2 Infection following incomplete or complete abortion

#### 2.2.1 Level 1 (Highest level, gold standard diagnosis, maximum sensitivity and specificity)

**Table:** 

Completed abortion or evidence of nonviable intrauterine pregnancy with gestational age within predefined range for selected abortion definition as assessed by maternal and/or fetal parameters (Level 1) (using the GAIA-Brighton Spontaneous Abortion and Ectopic Pregnancy Guidelines) within 42 days
AND
Measured fever to 38 degrees Celsius
AND
Evidence of uterine infection based on clinical examination (e.g., uterine tenderness, purulent vaginal discharge, prolonged vaginal bleeding, or presence of retained products of conception)
AND
Exclusion of urinary tract infection by negative urine culture or negative urine culture

#### 2.2.2 Level 2 (Missing at least one confirmatory diagnostic parameter, remains sensitive and specific)

**Table:** 

2A
Completed abortion or evidence of nonviable intrauterine pregnancy with gestational age within predefined range for selected abortion definition as assessed by maternal and/or fetal parameters (Level 2) (using the Brighton Spontaneous Abortion and Ectopic Pregnancy Guidelines) within 42 days
AND
Measured fever to 38 degrees Celsius
AND
Evidence of uterine infection based on clinical exam
AND
Exclusion of urinary tract infection by negative urine culture or negative urine culture
2B
Completed abortion or evidence of nonviable intrauterine pregnancy with gestational age within predefined range for selected abortion definition as assessed by maternal and/or fetal parameters (Level 1–2) (using the Brighton Spontaneous Abortion and Ectopic Pregnancy Guidelines) within 42 days
AND
Patient report of fever
AND
Evidence of uterine infection based on clinical exam
OR
Measured fever to 38 degrees Celsius
AND
Patient report of evidence of uterine infection
AND
Exclusion of urinary tract infection by negative urine culture or negative urine culture

#### 2.2.3 Level 3. (Less sensitive, with specificity)

**Table:** 

3A
Completed abortion or evidence of nonviable intrauterine pregnancy with gestational age within predefined range for selected abortion definition as assessed by maternal and/or fetal parameters (Level 3) (using the Brighton Spontaneous Abortion and Ectopic Pregnancy Guidelines) within 42 days
AND
Measured fever to 38 degrees Celsius
AND
Evidence of uterine infection based on clinical exam
OR
Patient report of fever
AND
Evidence of uterine infection based on clinical exam
OR
Measured fever to 38 degrees Celsius
AND
Patient report of evidence of uterine infection
AND
Exclusion of urinary tract infection by negative urine culture or negative urine culture
3B
Completed abortion or evidence of nonviable intrauterine pregnancy with gestational age within predefined range for selected abortion definition as assessed by maternal and/or fetal parameters (any level) (using the Brighton Spontaneous Abortion and Ectopic Pregnancy Guidelines) within 42 days
AND
Patient report of fever
AND
Evidence of uterine infection based on clinical exam
OR
Measured fever to 38 degrees Celsius
AND
Patient report of evidence of uterine infection
OR
Patient report of fever
AND
Patient report of evidence of uterine infection

## 3 Guidelines for data collection, analysis and presentation of postpartum endometritis and infection following incomplete or complete abortion

It was the consensus of the Brighton Collaboration *Postpartum Endometritis and Infection or Sepsis following Complete or Incomplete Abortion Working Group* to recommend the following guidelines to enable meaningful and standardized collection, analysis, and presentation of information. However, implementation of all guidelines might not be possible in all settings. The availability of information may vary depending upon resources, geographical region, and whether the source of information is a prospective clinical trial, a post-marketing surveillance or epidemiological study, or an individual report of postpartum endometritis or infection following incomplete or complete abortion. Also, as explained in more detail in the overview paper in this volume, these guidelines have been developed by this working group for guidance only, and are not to be considered a mandatory requirement for data collection, analysis, or presentation.

### 3.1 Data collection

These guidelines represent a desirable standard for the collection of available pregnancy outcome data following immunization to allow comparability. The guidelines are not intended to guide the primary reporting of abortion to a surveillance system. Investigators developing a data collection tool based on these data collection guidelines also need to refer to the criteria in the case definition, which are not repeated in these guidelines.

Guidelines 1–46 below have been developed to address data elements for the collection of adverse event information as specified in general drug safety guidelines by the International Conference on Harmonization of Technical Requirements for Registration of Pharmaceuticals for Human Use [ICHTR doc], and the form for reporting of drug adverse events by the Council for International Organizations of Medical Sciences [CIOMS]. These data elements include an identifiable reporter and patient, one or more prior immunizations, and a detailed description of the adverse event, in this case, of abortion following immunization. The additional guidelines have been developed as guidance for the collection of additional information to allow for a more comprehensive understanding of abortion following immunization.

#### 3.1.1 Source of information/reporter

For all cases and/or all study participants, as appropriate, the following information should be recorded:

(1) Date of report.
(2) Name and contact information of person^2^ reporting postpartum endometritis or infection following incomplete or complete abortion as specified by country specific data protection law.
(3) Relationship of the reporter to the vaccine recipient [e.g., immunizer (clinician, nurse) attending physician, family member [indicate relationship], self [vaccine recipient], other.

#### 3.1.2 Vaccinee/control

##### 3.1.2.1 Demographics

For all cases and/or all study participants (i.e. pregnant women), as appropriate, the following information should be recorded:

(4) Case study participant identifiers (first name initial followed by last name initial) or code (or in accordance with country- specific data protection laws).
(5) Date of birth, age of patient
(6) Gestational age at event or number of days postpartum
(7) Country of residence
(8) Occupation(s)

##### 3.1.2.2 Clinical and immunization history

For all cases and/or all study participants, as appropriate, the following information should be recorded:

(9) Past medical history, including hospitalizations, underlying diseases/disorders, pre-immunization signs and symptoms including identification of indicators for, or the absence of, a history of allergy to vaccines, vaccine components or medications; food allergy; allergic rhinitis; eczema; asthma.
(10) Any medication history (other than treatment for the event described) prior to, during, and after immunization including prescription and non-prescription medication as well as medication or treatment with long half-life or long term effect (e.g. immunoglobulins, blood transfusion and immune-suppressants) or substance abuse (e.g. narcotics or other recreational drug, alcohol or smoking).
(11) Immunization history (i.e. previous immunizations and any adverse event following immunization (AEFI), in particular occurrence of abortion after a previous immunization.
(12) Clinical confirmation of pregnancy prior to maternal immunization.

#### 3.1.3 Details of the immunization

For all cases and/or all study participants, as appropriate, the following information should be recorded:

(13) Date and time of immunization(s).
(14) Description of all vaccine (s) onset of PPE or infection following abortion (name of vaccines, manufacturer, lot number, expiration date, multi or mono dose vial, volume (e.g. 0.25 Ml, 0.5 mL, etc.), dose number if part of series of immunizations against the same disease(s), and the manufacturer, lot number, and expiration date of any diluents used).
(15) The anatomical sites (including left or right side) of all immunizations (e.g. vaccine A in proximal left lateral thigh, vaccine B in left deltoid).
(16) Route and method of administration (e.g. intramuscular, intradermal, subcutaneous, and needle-free (including type and size), other injection devices).
(17) Needle length and gauge.
(18) If the immunization is part of:
– Routine immunization program
– Preventive mass immunization campaign
– Mass immunization campaign for outbreak response
– Domestic travel from nonendemic to endemic area
– International travel
– Occupational risk

#### 3.1.4 The adverse event

For all cases at any level of diagnostic certainty and for reported events with insufficient evidence, the criteria fulfilled to meet the case definition should be recorded.

Specifically document (if available):

(19) Clinical description of signs and symptoms of postpartum endometritis or infection following incomplete or complete abortion, and if there was medical confirmation of the event (i.e. patient seen by physician).
(20) Date/time of onset^3^, first observation^4^ and diagnosis^5^; as well as end of episode^6^ and final outcome^7^, if appropriate (e.g. if the event no longer meets the case definition of abortion at the lowest level of the definition).
(21) Concurrent signs, symptoms, exposures and diseases.
(22) Pregnancy details:
– Pregnancy details: date of last normal menstrual period, ultrasound examinations, antenatal care visits, pregnancy-related illnesses and complications.
– Results of ultrasound examinations, antenatal care visits, laboratory examinations, other clinical tests, surgical and/ or pathological findings and diagnosis preferable to perform at reliable and accredited laboratories. If more than one measurement of a particular parameters is taken and recorded, the value corresponding to the largest deviation from the expected normal value or range of parameter should be reported.
– Event details: specifically document (if available) mode of treatment (e.g. IV antibiotics, etc) and complications, if any (e.g. hemorrhage, sepsis, etc.).
(23) Measurement/testing
– Values and units of routinely measured parameters (e.g. temperature, blood pressure) – in particular those indicating the severity of the event;
– Method of measurement (e.g. type of thermometer, oral or other route, duration of measurement, etc.);
– Results of laboratory examinations, surgical and/or pathological findings and diagnoses if present.
(24) Treatment given for postpartum endometritis or infection following incomplete or complete abortion, especially specify what and dosing, if applicable.
(25) Outcome^6^ at last observation. Add descriptions if maternal death occurred. Also, for multiple gestation, if concomitant twin death occurred. For example:
– Recovery to pre- immunization health status
– Spontaneous resolution
– Ongoing treatment
– Persistence of the event
– Significant complications of treatment
– Death and description of any other outcome
(26) Objective clinical evidence supporting classification of the event as “serious”^8^ (i.e. results in death), for example, a pathology report.
(27) Exposures other than the immunization before and after immunization (e.g. trauma, induced, environmental) considered potentially relevant to the reported event.

#### 3.1.5 Miscellaneous/ general

(28) The duration of follow-up reported during the surveillance period should be predefined likewise. It should aim to continue to resolution of the event (i.e. the outcome of the pregnancy or postpartum period is captured).
(29) Methods of data collection should be consistent within and between study groups, if applicable.
(30) Follow-up of cases should attempt to verify and complete the information collected as outlined in data collection guidelines 1 to 27.
(31) Investigators of patients with postpartum endometritis or infection following incomplete or complete abortion should provide guidance to reporters to optimize the quality and completeness of information provided.
(32) Reports of these events should be collected throughout the study period regardless of the time elapsed between immunization and the adverse event. If this is not feasible due to the study design, the study periods during which safety data are being collected should be clearly defined.
(33) The duration of surveillance period for these events should be predefined where applicable (e.g., clinical studies or active follow up) based on:
– Biologic characteristics of the vaccines (e.g., live attenuated versus inactivated component vaccines).
– Biologic characteristics of the vaccine- targeted disease.
– Biologic characteristics of abortion, including patterns identified in previous trials (e.g. early- phase trials) and
– Biologic characteristics of the target population (e.g., nutrition, underlying disease like immunesuppressive illness).
(34) Methods of data collection should be consistent within and between study groups or surveillance systems, if applicable.

### 3.2 Data analysis

The following guidelines represent a desirable standard for analysis of data on postpartum endometritis and infection following incomplete or complete abortion to allow for comparability of data, and are recommended as an addition to data analyzed for the specific study question and setting.

(36) Reported events should be classified in one of the following five categories including the three levels of diagnostic certainty. Events that meet the case definition should be classified according to the levels of diagnostic certainty as specified in the case definition. Events that do not meet the case definition should be classified in the additional categories for analysis.

#### 3.2.1 Event classification in 5 categories^9^

##### 3.2.1.1 Event meets case definition

(1) Level 1: *Criteria as specified in the Postpartum Endometritis or Infection Following Incomplete Or Complete Abortion case definition*
(2) Level 2: *Criteria as specified in the Postpartum Endometritis or Infection Following Incomplete Or Complete Abortion case definition*
(3) Level 3: *Criteria as specified in the Postpartum Endometritis or Infection Following Incomplete Or Complete Abortion case definition*

##### 3.2.1.2 Event does not meet case definition

###### 3.2.1.2.1 Additional categories for analysis

(4) Reported abortion with insufficient evidence to meet the case definition^10^
(5) Not a case of postpartum endometritis or infection following incomplete or complete abortion^11^
  (37) The interval between immunization and reported event could be defined as the date/time of immunization (last vaccination) to the date/time of onset^2^ of the event, consistent with the definition. If few cases are reported, the concrete time course could be analyzed for each; for a large number of cases, data can be analyzed in the following increments for identification of temporal clusters:

#### 3.2.2 Subjects with postpartum endometritis or infection following incomplete or complete abortion by interval to presentation

**Table:** 

Interval*	Number
≤ 24 hrs. after immunization	
2 - ≤ 7 days after immunization	
8 - ≤ 42 days after immunization	
> 42 days after immunization	
Weekly unit increments thereafter	

#### 3.2.3 Total

(38) If more than one measurement of a particular criterion is taken and recorded, the value corresponding to the greatest magnitude of the adverse experience could be used as the basis for analysis. Analysis may also include other characteristics like qualitative patterns of criteria defining the event.
(39) The distribution of data (as numerator and denominator data) could be analyzed in predefined increments (e.g. measured values, times), where applicable. Increments specified above should be used. When only a small number of cases is presented, the respective values or time course can be presented individually.
(40) Data on postpartum endometritis or infection following incomplete or complete abortion obtained from subjects receiving a vaccine should be compared with those obtained from an appropriately selected and documented control group(s) to assess background rates in non-exposed populations, and should be analyzed by study arm and dose where possible, e.g. in prospective clinical trials.

### 3.3 Data presentation

These guidelines represent a desirable standard for the presentation and publication of data on postpartum endometritis or infection following incomplete or complete abortion following immunization to allow for comparability of data, and are recommended as an addition to data presented for the specific study question and setting. Additionally, it is recommended to refer to existing general guidelines for the presentation and publication of randomized controlled trials, systematic reviews, and meta-analyses of observational studies in epidemiology (e.g. statements of Consolidated Standards of Reporting Trials (CONSORT), of Improving the quality of reports of meta-analyses of randomized controlled trials (QUORUM), and of Meta-analysis Of Observational Studies in Epidemiology (MOOSE), respectively) [Consort 2001, Moher 1999, Stroup 2000].

(41) All reported events of postpartum endometritis or infection following incomplete or complete abortion should be presented according to the categories listed in guideline 33.
(42) Data on possible events should be presented in accordance with data collection guidelines 1–32 and data analysis guidelines 33–37.
(43) Data should be presented with numerator and denominator (n/N) (and not only in percentages), if available.

Although immunization safety surveillance systems denominator data are usually not readily available, attempts should be made to identify approximate denominators. The source of the denominator data should be reported and calculations of estimates be described (e.g. manufacturer data like total doses distributed, reporting through Ministry of Health, coverage/population based data, etc.).

(44) The incidence of cases in the study population should be presented and clearly identified as such in the text.
(45) If the distribution of data is skewed, median and inter-quartile range are usually the more appropriate statistical descriptors than a mean. However, the mean and standard deviation should also be provided.
(46) Any publication of data on abortion should include a detailed description of the methods used for data collection and analysis as possible. It is essential to specify:
  • The study design;
  • The method, frequency and duration of monitoring for abortion;
  • The trial profile, indicating participant flow during a study including drop-outs and withdrawals to indicate the size and nature of the respective groups under investigation;
  • The type of surveillance (e.g. passive or active surveillance);
  • The characteristics of the surveillance system (e.g. population served, mode of report solicitation);
  • The search strategy in surveillance databases;
  • Comparison group(s), if used for analysis;
  • The instrument of data collection (e.g. standardized questionnaire, diary card, report form);
  • Whether the day of immunization was considered “day one” or “day zero” in the analysis;
  • Whether the date of onset^2^ and/or the date of first observation^3^ and/or the date of diagnosis^4^ was used for analysis; and
  • Use of this case definition for abortion, in the abstract or methods section of a publication^12^.

## 4 Disclaimer

The findings, opinions and assertions contained in this consensus document are those of the individual scientific professional members of the working group. They do not necessarily represent the official positions of each participant’s organization. Specifically, the findings and conclusions in this paper are those of the authors and do not necessarily represent the views of their respective institutions.

## Declaration of Competing Interest

The authors declare that they have no known competing financial interests or personal relationships that could have appeared to influence the work reported in this paper.

## Footnotes

9 To determine the appropriate category, the user should first establish, whether a reported event meets the criteria for the lowest applicable level of diagnostic certainty, e.g. Level three. If the lowest applicable level of diagnostic certainty of the definition is met, and there is evidence that the criteria of the next higher level of diagnostic certainty are met, the event should be classified in the next category. This approach should be continued until the highest level of diagnostic certainty for a given event could be determined. Major criteria can be used to satisfy the requirement of minor criteria. If the lowest level of the case definition is not met, it should be ruled out that any of the higher levels of diagnostic certainty are met and the event should be classified in additional categories four or five.

## Footnotes

2 If the reporting center is different from the vaccinating center, appropriate and timely communication of the adverse event should occur.

3 The date and/or time of onset is defined as the time post immunization, when the first sign or symptom indicative for abortion occurred. This may only be possible to determine in retrospect.

4 The date and/or time of first observation of the first sign or symptom indicative for abortion can be used if date/time of onset is not known.

5 The date of diagnosis of an episode is the day post immunization when the event met the case definition at any level.

6 The end of an episode is defined as the time the event no longer meets the case definition at the lowest level of the definition.

7 Example: recovery to pre-immunization health status, spontaneous resolution, therapeutic intervention, persistence of the event, sequelae, death.

8 An AEFI is defined as serious by international standards if it meets one or more of the following criteria: 1) it results in death, 2) is life-threatening, 3) it requires inpatient hospitalisation or results in prolongation of existing hospitalisation, 4) results in persistent or significant disability/incapacity, 5) is a congenital anomaly/birth defect, 6) is a medically important event or reaction. For abortion, the event meets the definition of serious (i.e. it results in death of the embryo/fetus).

10 If the evidence available for an event is insufficient because information is missing, such an event should be categorised as “reported abortion with insufficient evidence to meet the case definition”.

11 An event does not meet the case definition if investigation reveals a negative finding of a necessary criterion (necessary condition) for diagnosis. Such an event should be rejected and classified as “Not a case of abortion”.

12 Use of this document should preferably be referenced by referring to the respective link on the Brighton Collaboration website (http://www.brightoncollaboration.org).
